# Aggressive Primary Cutaneous Apocrine Carcinoma of the Scalp: A Clinical Case Report

**DOI:** 10.7759/cureus.77444

**Published:** 2025-01-14

**Authors:** Elaheh Shaghaghian, David J Park, Krishna L Bharani, Subhro K Sen, Steven D. Chang

**Affiliations:** 1 Department of Neurosurgery, Stanford University School of Medicine, Palo Alto, USA; 2 Department of Pathology, Stanford University School of Medicine, Palo Alto, USA; 3 Department of Plastic Surgery, Stanford University School of Medicine, Palo Alto, USA

**Keywords:** aggressive, apocrine glands, carcinoma, neurosurgery, pathology, scalp

## Abstract

Primary cutaneous apocrine carcinoma (PCAC) is a rare malignancy originating from the apocrine glands of the skin. It is predominantly found in regions rich in apocrine glands, such as the axilla and anogenital area. Scalp involvement is rare, and the disease's aggressive behavior further complicates its management.

A 78-year-old male with a history of primary cutaneous apocrine carcinoma (PCAC) of the neck, previously managed with craniotomy, radiation, and chemotherapy, presented five years later with recurrence and bone metastasis. Six years after the initial diagnosis, he developed enlarging masses in the neck, necessitating extensive surgical resection. Histopathology confirmed primary cutaneous cribriform apocrine carcinoma with positive markers for CK7, GCDFP-15, EMA, and androgen receptors. Further molecular analysis performed on a fine-needle aspiration biopsy revealed pathogenic and likely pathogenic alterations, including HRAS G13R and FBXW7 Y545C. The patient underwent successful resection of the neck apocrine carcinoma by neurosurgeons, followed by soft tissue reconstruction by plastic surgeons.

This case highlights the importance and challenges of diagnosing and managing a scalp PCAC with recurrence and bone metastasis and the need for more aggressive follow-up and treatment strategies in similar cases.

## Introduction

Primary cutaneous apocrine carcinoma (PCAC) is an uncommon malignancy originating from the apocrine glands of the skin, especially seen in regions rich in apocrine glands, such as the axilla and anogenital area, although cases involving the extremities have also been documented in the medical literature [1,2]. While scalp involvement is infrequent, cases demonstrating aggressive behavior present unique challenges in diagnosis and management. Mainly seen in the elderly population, PCAC manifests as an indurated dermal or subcutaneous plaque, often presenting as painless, solitary, or multifocal masses [3]. Although its exact etiology remains unclear, PCAC predominantly affects individuals in their sixth decade of life, with the Caucasian population exhibiting a higher predisposition [1,3,4]. Histologically, PCAC is characterized by a range of architectural patterns, including tubular, cribriform, and solid growth, with cytological atypia and decapitation secretion being key features. Due to the rarity of this tumor, a definitive diagnostic immunohistochemical profile has not been well established; however, markers such as cytokeratins (CK), gross cystic disease fluid protein (GCDFP-15), and androgen receptors can help delineate apocrine differentiation [3].

Thus, in this presented case, a 78-year-old male with multiple recurrent apocrine carcinomas in the neck extending to the scalp underwent surgical intervention due to the aggressive nature of the disease. A team of neurosurgeons and plastic surgeons performed the resection, followed by soft tissue reconstruction using free tissue transfer, preserving neurovascular structures and ensuring clear margins.

## Case presentation

**Figure 1:**
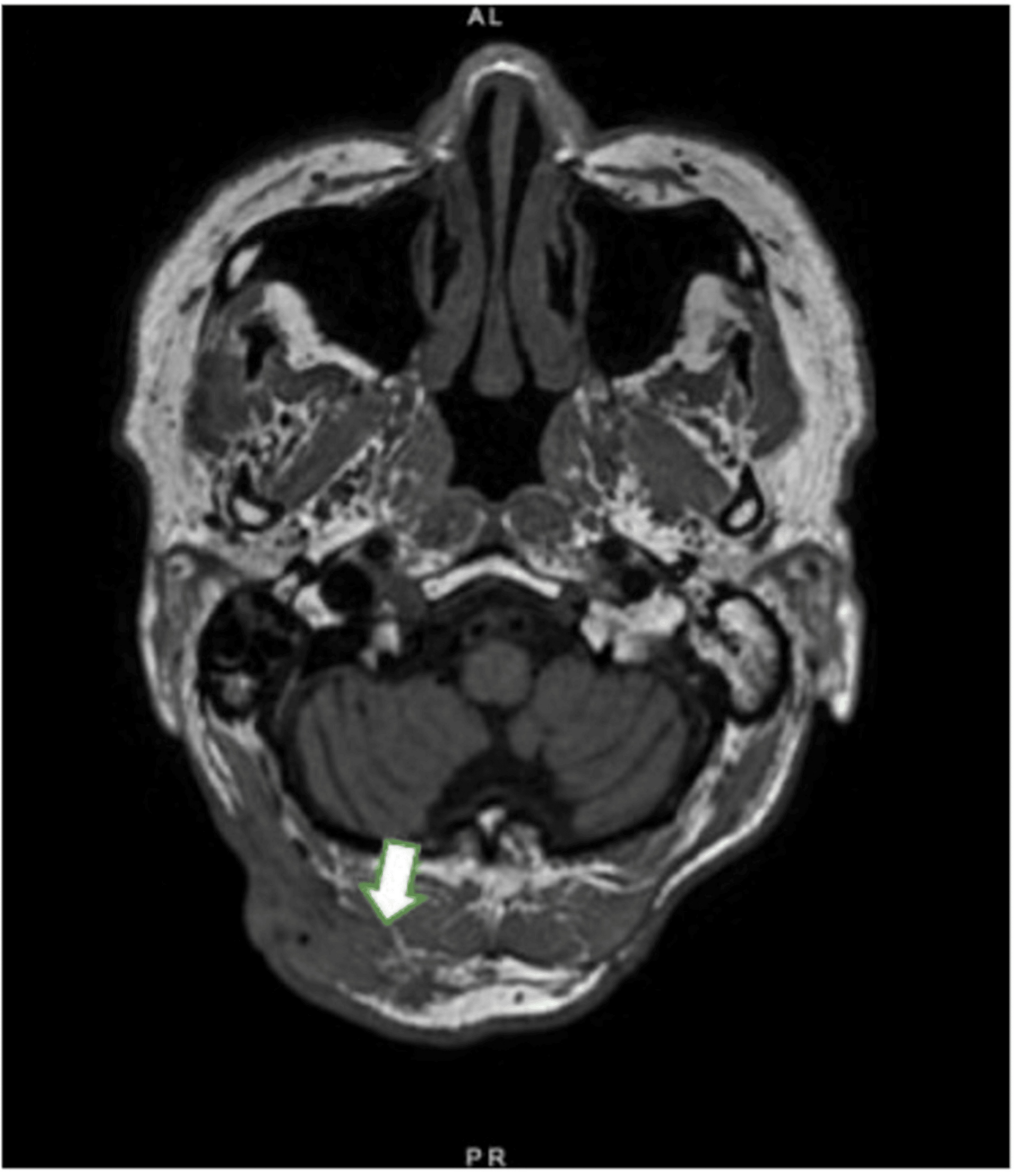
Suboccipital soft tissue mass The arrow in T1 magnetic resonance imaging (MRI) points to the 2.1 x 1.6 x 4.6 cm irregular enhancing right suboccipital soft tissue mass infiltrating the fascia and abutting the right splenius capitis and rectus capitis.

**Figure 2:**
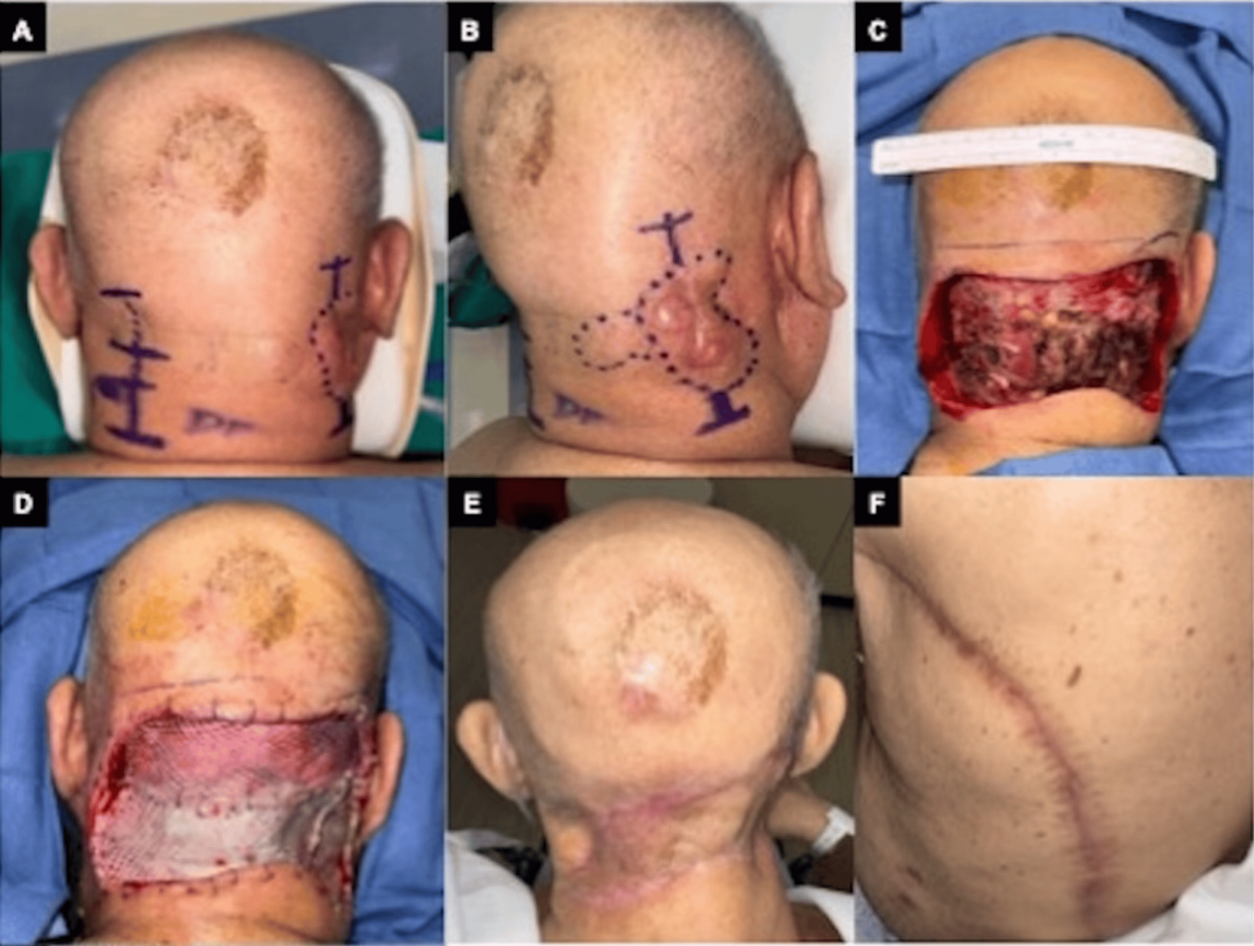
The preoperative and postoperative images of apocrine carcinoma of the scalp A, B: the preoperative image captures the presentation of a 78-year-old male with bilateral stage IV apocrine carcinoma of the scalp, exhibiting a painless, reddish ulcer; C, D: intraoperative snapshot displays meticulous excision of the scalp mass, excision of the open wound of the posterior neck, 25 sq cm; E, F: post-treatment figures.

A 78-year-old male first presented with a dome-shaped, nonpainful nevus on the occipital region of the neck that had changed from an alopecic patch since birth. For many years, the lesion did not increase in size and was not symptomatic until six years ago, when it started to gradually grow in size and occasionally bleed, ultimately leading to surgical removal (Figures 1, 2). 

**Figure 3:**
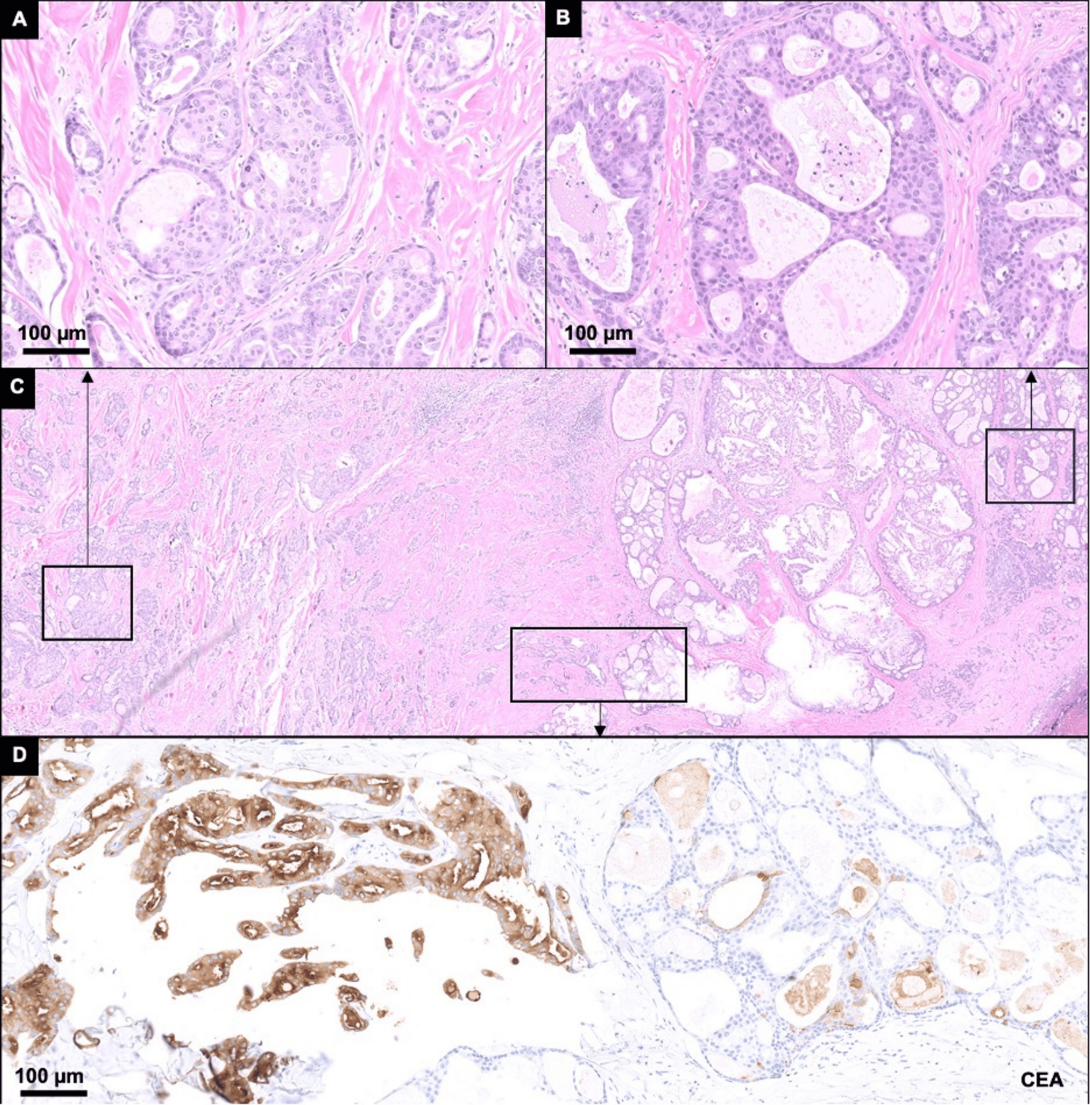
Histological and cytological features of the initial resection Histology from the patient’s initial resection with two proliferations with distinct histology. The first proliferation is composed of atypical epithelioid cells with ovoid to angulated nuclei forming tubular to cribriform architecture A. A: at low power, H&E-stained histologic sections from the right neck mass resection show a lesion invading the dermis; B: perineural invasion; C: mitotic figures are present; D: by immunohistochemistry, the atypical cells are positive for androgen receptor.

An initial surgical excision of this lesion revealed two proliferations with distinct histology arising from a background nevus sebaceous on routine hematoxylin and eosin (H&E) stained sections (Figure 3).

The first proliferation was composed of atypical epithelioid cells forming cystic and nodular lesions with cribriform architecture that was focally connected with the epidermis and invaded the dermis. Scattered mitotic figures and decapitation secretions were appreciated, and definitive perineural invasion was not identified. Considering the histologic features, the differential diagnosis comprised primary cutaneous cribriform apocrine carcinoma, adenoid cystic carcinoma, adenoid cystic basal cell carcinoma, syringoid eccrine carcinoma, and metastatic carcinoma. By using immunohistochemistry, the first proliferation expressed positive for CEA, EMA, and CK7, negative for S100 and CD117, and mostly negative for BEREP4, supporting the diagnosis of primary cutaneous apocrine carcinoma. The overall immunoprofile of the PCAC in this patient was positive for androgen receptor (AR), CK7, CEA (focal), BERP4 (focal), EMA, GATA3 (patchy), and GCDFP-15 and negative for calponin, CD117, CK5, CK20, HER2 (equivocal) ER, p63, PR, S100, and SMA. The second proliferation was immediately adjacent to the first and was composed of basaloid keratinocytes forming cribriform architecture with cystic spaces in well-demarcated nodules and nests. Stomal clefts, necrotic cells, and mitotic figures were identified. By immunohistochemistry, the second proliferation was notably positive for BEREP4 and negative for CEA, which led to the diagnosis of nodular basal cell carcinoma. These histologic findings supported the diagnosis of a PCAC and basal cell carcinoma (BCC) arising from a nevus sebaceous. Accordingly, the patient underwent surgical craniotomy resection of the tumor and local radiotherapy.

Despite initial treatment, the patient experienced a recurrence of the apocrine carcinoma in the occipital region four years later. The subsequent surgical resection revealed similar morphology as the first proliferation found in the initial biopsy. One year after this occipital resection, the patient experienced a fall resulting in a left hip lytic lesion. Pathological examination revealed metastatic adenocarcinoma and marrow involvement (90% involvement of marrow by metastatic cancer), consistent with PCAC. The patient subsequently underwent open reduction and internal fixation (ORIF), and he was diagnosed with metastatic adenocarcinoma to the left femur. He was placed on chemotherapy, which continued through the end of that year with carboplatin/taxol for stage IV apocrine carcinoma of the scalp with biopsy-proven bone metastasis.

**Figure 4:**
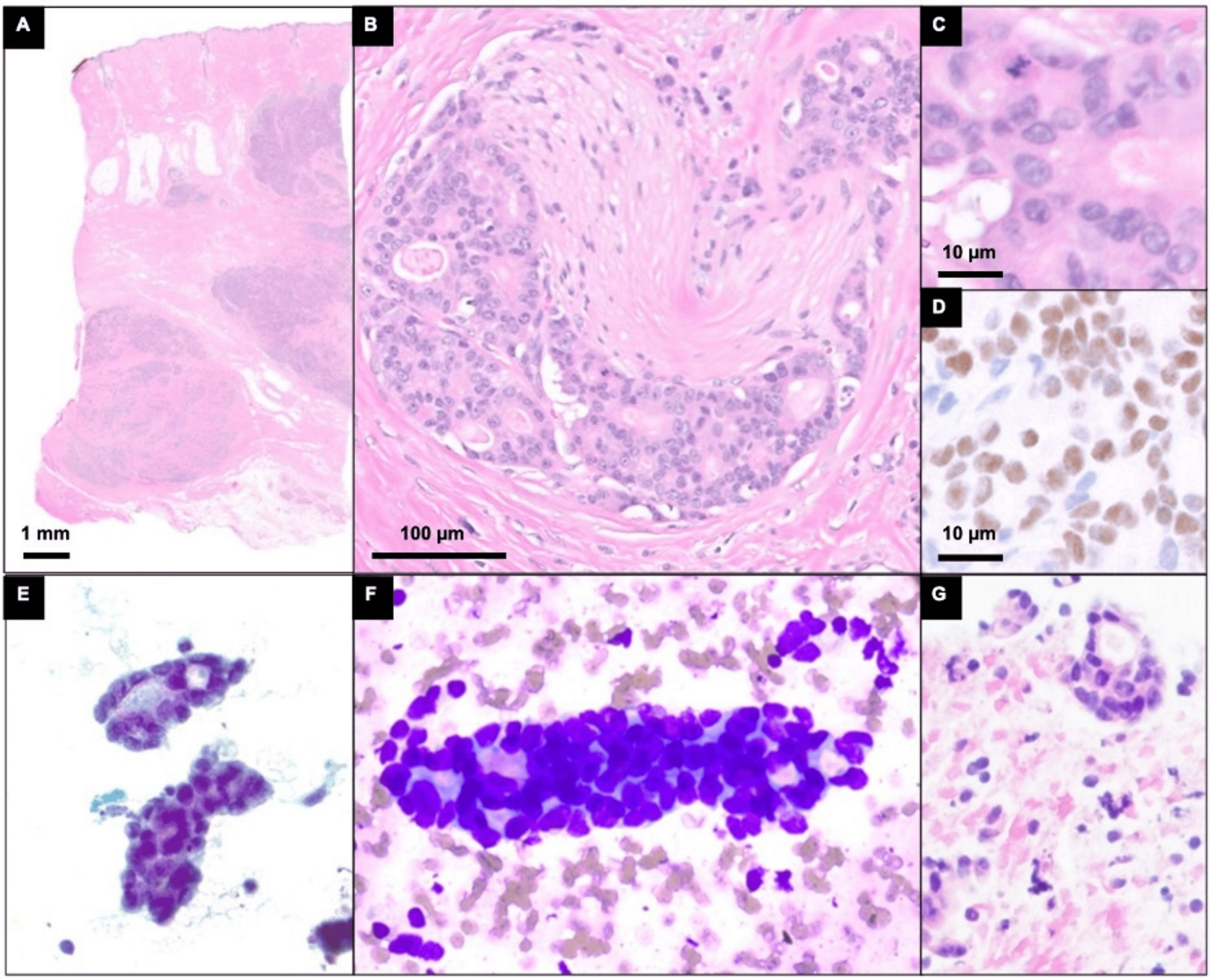
Histologic and cytologic features of right neck mass A: at low power, H&E-stained histologic sections from the right neck mass resection show a lesion invading the dermis; B: perineural invasion; C: mitotic figures are present; D: by immunohistochemistry, the atypical cells are positive for androgen receptor; E: aspirate smears show clusters of carcinoma cells with ovoid nuclei, nuclear overlapping, and variably prominent nucleoli at high power on Papanicolaou staining; and F: Romanowsky staining; G: cell block preparations with H&E staining show atypical epithelioid cells with ovoid to angulated nuclei forming clusters and tubules.

One year after the bone metastasis diagnosis, further progression of apocrine carcinoma on both the left and right sides of the neck required additional surgical intervention. Over time, the lesion exhibited gradual enlargement and intermittent bleeding, culminating in a 2.1 x 1.6 x 4.6 cm painless ulcerative growth with a verrucous periphery (Figure 2). A fine-needle aspirate (FNA) biopsy of this lesion was performed for molecular analysis and displayed atypical epithelioid cells with similar morphology as the patient's prior excisions (Figure 4). 

Next-generation sequencing-based mutational profiling using the Stanford Actionable Mutation Panel (STAMP) for solid tumors, version 4.3.2, performed on the FNA biopsy, identified alterations HRAS G13R (VAF 70%, pathogenic) and FBXW7 Y545C (VAF 32%, likely pathogenic). In addition, the following variants of unknown significance were identified: ARID1A S128T, CASP8 c.-27+24192C>T, DICER1 D929G, ERBB4 H811Y, FGFR2 V12M, MTOR T1870S, MYCN R170P, and SMARCA4 Q201L. The invasive carcinoma was surgically removed, and histopathological examination confirmed primary cutaneous cribriform apocrine carcinoma.

Overall, the specimens available for histopathologic evaluation included the initial surgical excision of the posterior neck/scalp lesion, surgical excision of the occipital metastasis five years from the initial presentation, fine-needle aspirate (FNA) biopsies, and surgical excision of the occipital lesion recurrence six years from the initial presentation, and a bone marrow biopsy. The metastatic specimens showed similar morphology as the first proliferation in the initial biopsy and were positive for CK7 and androgen receptors and negative for BERP4, which supported the diagnosis of metastatic PCAC. Evidence of basal cell carcinoma that was present in the initial resection was not identified in the metastatic specimens. Notably, in comparison to the initial PCAC, the metastatic PCAC had prominent perineuronal invasion (Figure 4). 

The patient's treatment course included the excision of invasive apocrine carcinoma in the occipital and cervical regions. Right and left neck apocrine carcinoma resections were performed, and the resulting wound (Figure 2D) was temporized with a cadaveric akin allograft (Figure 2E). Once final, negative pathologic margins were obtained, staged soft tissue reconstruction was performed by the plastic surgeons to close the wound using a left latissimus dorsi muscle free flap and split-thickness skin graft from the left thigh (Figure 2).

The patient’s early postoperative course following the resection of the occipital tumor and free tissue transfer was uncomplicated. However, at the first postoperative follow-up visit, it was discovered that there was marginal skin necrosis and wound dehiscence at the left back flap donor site. The patient was then taken back to surgery, where eschar excision, seroma drainage, and secondary closure were performed. The patient recovered well from the procedure without complications, and all surgery sites healed well.

This paper focuses on the complex management issues arising from the case of advanced apocrine carcinoma of the scalp and provides cytologic and molecular features of this lesion. Multidisciplinary collaboration was pivotal in navigating this complex malignancy, underscoring the importance of ongoing surveillance to monitor disease progression and guide further therapeutic interventions. Thus, it was critical in the management of this complex malignancy to maintain multidisciplinary cooperation. Further research and clinical investigations are mandatory to enhance the current therapeutic approach and individual patients’ outcomes in this rare and challenging disease.

## Discussion

Primary cutaneous apocrine carcinoma (PCAC) is a rare skin tumor that raises diagnostic and management difficulties because of the tumor’s high anaplastic grade and its capacity to metastasize [5,6]. According to the literature, only about 200 cases have been reported, with the origin site mostly being the axillary area because of its richness in apocrine glands. Scalp involvement, on the other hand, is rare across the cases and rarely contributes to more than a fraction. On the other hand, scalp involvement is relatively rare and forms a minority of reported cases [5]. Our case illustrates these issues as the patient was a 78-year-old male with bilateral stage IV PCAC of the scalp and biopsy-proven bone metastasis. The patient initially presented six years ago with apocrine carcinoma treated via craniotomy, local radiation, and chemotherapy. This was followed by a recurrence and subsequent additional craniotomy, highlighting the aggressive and recurrent nature of PCAC. The case was further complicated by the presence of a long-standing dome-shaped tumor on the scalp that evolved from an alopecic patch present since birth, eventually developing into a significant ulcerative lesion.

Histopathologic evaluation of the tumor revealed two distinct infiltrating proliferations and initially invoked diagnostic considerations for cystic carcinoma with ductal differentiation, including PCAP, adenoid cystic carcinoma, adenoid cystic basal cell carcinoma, and syringoid eccrine carcinoma, as well as metastatic carcinoma. The immunohistochemical profile in combination with the histology ultimately helped favor the diagnosis of PCAC in one proliferation and nodular BCC in the adjacent lesion. Commonly positive markers in PCAC included CEA, EMA, CK7, GCDFP-15, and GATA3, while negative markers encompassed CK5/6, CK20, S100, CD117, and calponin; however, these markers are not specific for PCAC, and their evaluation must be approached holistically, considering the differential diagnoses. Robson et al. (2008) highlighted the utility of hormone receptor staining in diagnosing PCAC with a significant proportion of cases expressing estrogen and androgen receptors [7,8]. Our case demonstrated positive androgen receptor staining consistent with these findings, although estrogen receptor expression was absent. This variability underscores the heterogeneity of PCAC and the need for comprehensive immunohistochemical profiling in its diagnosis [8].

Table 1 describes twelve cases of apocrine carcinoma of the scalp and presents the patient demographics, tumor characteristics, treatment outcomes, and immunohistochemistry profiles. The patients were between the ages of 45 to 78 years, with a median age of 66 years, and seven males and four females, with one case not reporting sex. Tumor sizes ranged from 0.7 cm to 7 cm, which was more common in the scalp region. The growth periods of the tumors were highly variable, from congenital lesions to growth over several months or years. Notably, two cases reported coexistent tumors, specifically Syringocystadenoma Papilliferum, and trichoblastoma, highlighting the potential multifocal nature and complexity of these lesions (Table 1).

**Table 1:** . Clinical data of 12 case reports of primary cutaneous apocrine carcinoma arising from nevus sebaceous M: male; F: female; LN: lymph node; LR: local recurrence; AWD: alive with disease; NED: no evidence of disease; LTF: lost to follow-up; DWD: died with disease; NR: not reported; EMA: epithelial membrane antigen; CEA: carcinoembryonic antigen; GCDFP-15: gross cystic disease fluid protein 15; HER-2: human epidermal growth factor receptor 2 This table summarizes historical cases from the literature, with our case data included as an addition.

Number	Reference	Age (уг)	Sex	Size (cm)	Time evolved, growth	Location	Coexistent tumor	Recurrence or metastases	Outcome (follow-up)	Immunohistochemistry profile
1	Kim et al., 2023 [3]	59	M	3.3×2.1×1.5	Unknown 1 yr growth	Scalp	Syringo- cystadenoma papilliferum	NED	NED (6 mon)	GATA3(+), GCDFP-15(+), AR (+), CK5/6(–)
2	Robson et al., 2008 [7]	NR	NR	NR	NR	Scalp	NR	NR	NR	-
3	Domingo and Helwig, 1979 [9]	77	M	2x1.2	1.5 mon	Scalp	-	LN cervical (6 mon) LR (1.5 yr)	AWD (1.5 yr)	-
4	Domingo and Helwig, 1979 [9]	63	F	1.5	Birth	Scalp	-	NED	NED (6 yr)	-
5	Domingo and Helwig, 1979 [9]	68	F	0.7	Unknown	Scalp	-	NR	LTF	-
6	Domingo and Helwig, 1979 [9]	65	M	7	Birth, 1 mon growth	Scalp	-	LN post-auricular, cervical, and supraclavicular (6 mon) metastasis, bone (9 mon) DWD (2 yr)	DWD (2 yr)	-
7	Jacyk et al., 1998 [10]	53	F	4x1	Since childhood, few months growth	Scalp	Syringo- cystadenoma papilliferum	NED	NED (1 yr)	MNF-116 (+)/ luminal border: EMA (+), CEA (+), GCDFP-15(+)
8	Dalle et al., 2003 [11]	66	M	0.8	Birth, 6 mon growth	Scalp	-	NR	NR	CEA (+), EMA (+), CK7(+), CD15 (Leu-M1) (+)
9	Tanese et al., 2010 [12]	70	M	2	2 mon	Scalp	-	NR	NR	p53(+), HER-2(+), ER (-), PR-), AR (-)
10	Paudel et al., 2012 [13]	45	M	2x2	Since childhood, 4 mon growth	Scalp	-	NR	NR	-
11	Edgar et al., 2018 [14]	76	F	2.5	2 mon	Scalp	Trichoblastoma	NED	NED (10 mon)	CEA (+), EMA(+), CK7(+)
12	Shaghaghian et al. Current case report	78	M	2.1 x 1.6 x 4.6	Birth, 6 yr growth	Scalp	-	LR (6 years), Metastasis to bone (5years)	AWD (6 years)	GATA3(+), GCDFP-15(+), AR (+), EMA (+), CK7(+), CK5/6(–), CK20(–) , S100(–) , CD117(–), Calponin(–)

While the literature documents the association of nevus sebaceous with various neoplasms, including BCC, the simultaneous presence of PCAC and BCC basal cell carcinoma is rare. This case report adds to the limited literature suggesting multiple neoplasms can arise from a nevus sebaceous, particularly in older patients.

This case report also adds to the limited genomic analysis of PCAC, which, if better understood, could reveal novel therapeutic avenues. Next-generation sequencing on PCAC, in our case, identified alterations HRAS G13R and FBXW7Y545C. HRAS G13R is a known oncogenic mutation and is associated with the activation of MAPK and PI3K signaling, leading to cell proliferation. RAS mutations are acknowledged pathogenic genomic alterations in sebaceous nevus, which are preserved in secondary tumors, and more specifically, HRAS G13R has been described in apocrine carcinoma and is consistent with PCAC diagnosis arising from a sebaceous nevus in this report [15]. FBXW7 is among the family of F-box proteins that are involved in the ubiquitination and destruction through the proteasome degradation of oncoproteins and about the FBXW7 Y545C missense mutation in breast invasive ductal carcinoma, head and neck squamous carcinoma, and uterine carcinoma. Loss of FBXW7 in other cancers (lung, colon, and hematopoietic) has been linked to resistance to chemotherapeutic agents and poorer disease outcomes [16], but its significance in PCAC remains unclear.

Recurrence and metastasis were significant concerns observed in several cases. For instance, case 3 [9] exhibited cervical lymph node metastasis within six months and local recurrence at 1.5 years, whereas case 6 [9] experienced lymph node metastasis at six months and bone metastasis at nine months, culminating in death within two years. The current case (case 12) showed metastasis to the bone at five years and cervical recurrence at six years, underscoring the aggressive behavior of apocrine carcinoma. On histology, the metastatic PCAC was identified by its distinct morphology and its immunopositivity to CK7 and androgen receptor. Notably, the focal BEREP4 positivity observed in the primary PCAC biopsy was absent in the metastatic lesions, which displayed prominent perineuronal invasion.

Despite aggressive therapeutic interventions, outcomes for patients with PCAC varied significantly; some patients achieved no evidence of disease (NED) after extended follow-ups (median follow-up: 1.5 years), while others either succumbed to the disease or remained alive with persistent disease (AWD). This analysis underscores the necessity of a multidisciplinary approach, regular follow-ups, and personalized treatment strategies to optimize outcomes for patients with advanced apocrine carcinoma of the scalp. Continued research and clinical insights are imperative to refine treatment paradigms and enhance patient care in this complex condition (Table 1). Standard therapy involves surgical excision, and the condition of the lymph nodes is the primary determinant of survival in early-stage disease. Sentinel lymph node biopsy may be considered in the treatment protocol for PCAC [17].

The patient’s extensive treatment history, including multiple resections, radiation, chemotherapy, and complex reconstructive surgeries, highlights the necessity of a multidisciplinary approach to managing advanced PCAC [2,4,18]. The use of a latissimus dorsi muscle-free flap and skin graft for scalp wound reconstruction exemplifies the critical role of plastic surgery in achieving optimal functional and aesthetic outcomes in such complex cases. Moreover, the presence of chronic lacunar infarcts and soft tissue masses on postoperative brain MRI, suggestive of nodal and soft tissue metastasis, highlights PCAC's aggressive behavior and the importance of vigilant postoperative surveillance. In our case, the tumor’s resilience may be linked to the FBXW7 missense mutation identified through next-generation sequencing, which has been associated with resistance to chemotherapy in other carcinomas. Overall, this case emphasizes the need for ongoing monitoring and a tailored therapeutic approach to manage disease progression effectively.

In conclusion, this case illustrates the intricate management challenges posed by the advanced PCAC of the scalp. The combination of comprehensive clinical evaluation, detailed histologic and molecular analysis, and multidisciplinary collaboration is crucial in navigating the complexities of this rare malignancy. Ongoing research and clinical insights are essential to refining treatment paradigms and improving patient outcomes in PCAC.

## Conclusions

This case highlights the significant difficulties in dealing with aggressive primary cutaneous apocrine carcinoma (PCAC) of the scalp, mainly when it is in recurrent disease. Surgical removal of tumors with clear margins is a marker of a successful operation in reducing the chances of local failure due to the presence of cancerous cells. Due to the infiltrative nature of PCAC, the preoperative planning must involve advanced imaging and careful assessment of the margin of the tumor, and then there must be careful intraoperative attention paid to make sure all areas of involvement are properly excised. Multidisciplinary collaboration is essential for optimizing patient outcomes. Because of the complex nature of PCAC, multidisciplinary teamwork of neurosurgeons, plastic surgeons, dermatologists, oncologists, and pathologists should be encouraged in order to deliver an adequate assessment to patients. Every specialty predicts an important role in diagnosis, staging, surgery performed with excision, reconstruction, and post-surgical follow-up. It is especially important in scalp reconstruction since functional and aesthetic outcomes are often challenging to achieve due to the incorporation of sensitive structures.

Moreover, this case demonstrates the value of molecular analysis for elucidating PCAC’s etiology and, potentially, the development of treatment strategies. The HRAS G13R and FBXW7 Y545C mutations detected by next-generation sequencing give much information about the tumor’s behavior and its immunity to the common treatments. It becomes apparent that molecular targets create new opportunities for treatment, especially in cases when conventional chemotherapy and surgery are ineffective in the treatment of PCAC. In conclusion, the optimal treatment of highly malignant PCAC depends upon accurate and meticulous surgery, the most efficient collaboration of various specialties, as well as the constant development of new approaches to the treatment of neoplastic disease. A better understanding of this rare cancer will enable the identification of the most effective and targeted therapies that will help decrease the rates of recurrences and increase the overall survival rate.
